# External validation study on the value of deep learning algorithm for the prediction of hematoma expansion from noncontrast CT scans

**DOI:** 10.1186/s12880-022-00772-y

**Published:** 2022-03-14

**Authors:** Dong Chuang Guo, Jun Gu, Jian He, Hai Rui Chu, Na Dong, Yi Feng Zheng

**Affiliations:** 1 Department of Radiology, Huzhou Central Hospital, Affiliated Central Hospital of Huzhou University, Huzhou, 313000 Zhejiang Province China; 2 Institute of Clinical Research, Biomind Technology, Beijing, 100050 China

**Keywords:** Artificial intelligence, Hypertensive intracerebral hemorrhage, Hematoma expansion, Early diagnosis

## Abstract

**Background:**

Hematoma expansion is an independent predictor of patient outcome and mortality. The early diagnosis of hematoma expansion is crucial for selecting clinical treatment options. This study aims to explore the value of a deep learning algorithm for the prediction of hematoma expansion from non-contrast computed tomography (NCCT) scan through external validation.

**Methods:**

102 NCCT images of hypertensive intracerebral hemorrhage (HICH) patients diagnosed in our hospital were retrospectively reviewed. The initial computed tomography (CT) scan images were evaluated by a commercial Artificial Intelligence (AI) software using deep learning algorithm and radiologists respectively to predict hematoma expansion and the corresponding sensitivity, specificity and accuracy of the two groups were calculated and compared. Comparisons were also conducted among gold standard hematoma expansion diagnosis time, AI software diagnosis time and doctors’ reading time.

**Results:**

Among 102 HICH patients, the sensitivity, specificity, and accuracy of hematoma expansion prediction in the AI group were higher than those in the doctor group(80.0% vs 66.7%, 73.6% vs 58.3%, 75.5% vs 60.8%), with statistically significant difference (*p* < 0.05). The AI diagnosis time (2.8 ± 0.3 s) and the doctors’ diagnosis time (11.7 ± 0.3 s) were both significantly shorter than the gold standard diagnosis time (14.5 ± 8.8 h) (*p* < 0.05), AI diagnosis time was significantly shorter than that of doctors (*p* < 0.05).

**Conclusions:**

Deep learning algorithm could effectively predict hematoma expansion at an early stage from the initial CT scan images of HICH patients after onset with high sensitivity and specificity and greatly shortened diagnosis time, which provides a new, accurate, easy-to-use and fast method for the early prediction of hematoma expansion.

Hypertensive intracerebral hemorrhage (HICH) refers to the sudden occurrence of cerebral parenchymal hemorrhage in patients with a clear history of hypertension, excluding secondary cerebral hemorrhage caused by trauma, vascular abnormalities, coagulation dysfunction, blood diseases and other diseases. HICH is the most common form of stroke accounting for about 50–70% of spontaneous intracerebral hemorrhage [1]. Spontaneous intracerebral hemorrhage (SICH) causes severe threats to patients physically and mentally, because it starts suddenly and worsens rapidly resulting in a high fatality and disability rate [2]. About 30–50% [3–5] SICH patients died within one month since disease onset, and about 1/3 of these patients had hematoma expansion, of which 73% had hematoma expansion within 24 h [6–8]. Hematoma expansion is an independent predictor of patient outcome and mortality. Therefore, early diagnosis of hematoma expansion is crucial for selecting clinical treatment options [9, 10].

Many imaging methods have been explored to predict hematoma expansion in patients with HICH. However, the sensitivity of predicting hematoma expansion remains low simply by analyzing these imaging features. For example, the spot sign of Computed Tomography Angiography (CTA) has been explored for hematoma expansion prediction in patients with HICH [11, 12] and several studies have shown that the sensitivity of CTA spot sign for the prediction of hematoma expansion is 26.2–73% [10, 13]. In one recent study, the sensitivity of CTA spot sign to predict hematoma expansion was merely 34.6% [14]. CTA scan increased radiation dose and contrast agent injection may lead to allergic reactions and renal damage. In addition, many primary hospitals lack medical resources and might not be able to carry out CTA examinations limiting the broad application of CTA [15–17]. Some scholars seek to predict hematoma expansion by analyzing imaging features from NCCT images in HICH patients and several studies have shown that the prediction sensitivity ranges from 31 to 55% [17–20]. In addition, the prediction of hematoma expansion through imaging features is subjective and the inter-reader agreement was merely moderate [16, 21]. Moreover, time is the brain, early and quick prediction of hematoma expansion significantly impacts on patient prognosis [22]. At present, hematoma expansion is generally determined by changes in hematoma volume of two CT scans within 24 h since hospital admission and the interval time is relatively long. Therefore, it is imperative to find an early, reliable and easy-to-use method for predicting hematoma expansion.

AI is gaining increased use in the diagnosis of brain diseases and one latest study shows that the sensitivity to detect intracranial aneurysms of radiologists could be improved from 62.86 to 86.67% with the assistance of a deep learning algorithm [23]. Wang Jiwen et al. applied deep learning to segment CT images to measure hematoma volume of 1223 patients with SICH and the resulting measurement of hematoma volume by deep learning was more accurate than the ABC/2 formula [24]. Sun Haiyue [25] established a decision tree model to predict 196 patients with HICH. It was found that the outcome prediction was in good agreement with the actual outcome, which could assist the clinical treatment of HICH. However, few studies used AI to predict hematoma expansion in HICH patients. One previous study reported a hematoma prediction AI model which provided a time-saving, easy to implement, and subjective and independent method to predict the risk of hematoma enlargement in patients with intracerebral hemorrhage based on the NCCT images [26]. This study aimed to explore the value of the deep learning algorithm for the prediction of hematoma expansion from NCCT scans through external validation.

## Materials and methods

### Patient characteristics

Brain CT images of HICH patients diagnosed in our hospital from January 2019 to October 2020 were retrospectively collected and analyzed. An initial CT scan was performed within 6 h of onset and a follow-up CT scan was performed within 24 h of admission. Exclusion criteria were: (1) Patients with a history of cerebral hemorrhage or had undergone brain surgery or had undergone brain surgery within 24 h after admission (2) Poor image quality that affects image evaluation. This study was reviewed and approved by the Institutional Review Board of our hospital.

### Scanning protocol

A conventional head scan was performed using 16-row spiral CT (Brilliance CT, Philips). Scanning parameters were: tube voltage120 kV, tube current 300mAs, slice thickness and increment were 4.5 mm, matrix 512 × 512.

### Gold standard for hematoma expansion

Hematoma volumes were manually measured by two radiologists (Doctor A, Doctor B) with 5 and 6 years of experience in diagnosing cerebral hemorrhage and calculated using ABC/2 formula. Hematoma expansion was determined if the hematoma volume change of the two CT scans within 24 h increased by more than 12.5 ml or 33% [18]. Hematoma expansion was reviewed by two doctors and referred by a senior neuroradiologist (Doctor C, 20 years of experience in diagnosing cerebral hemorrhage) to confirm the final diagnosis after his first review independently. The final diagnosis of the neuroradiologist was used as the gold standard for hematoma expansion.

### AI diagnosis

**Fig. 1:**
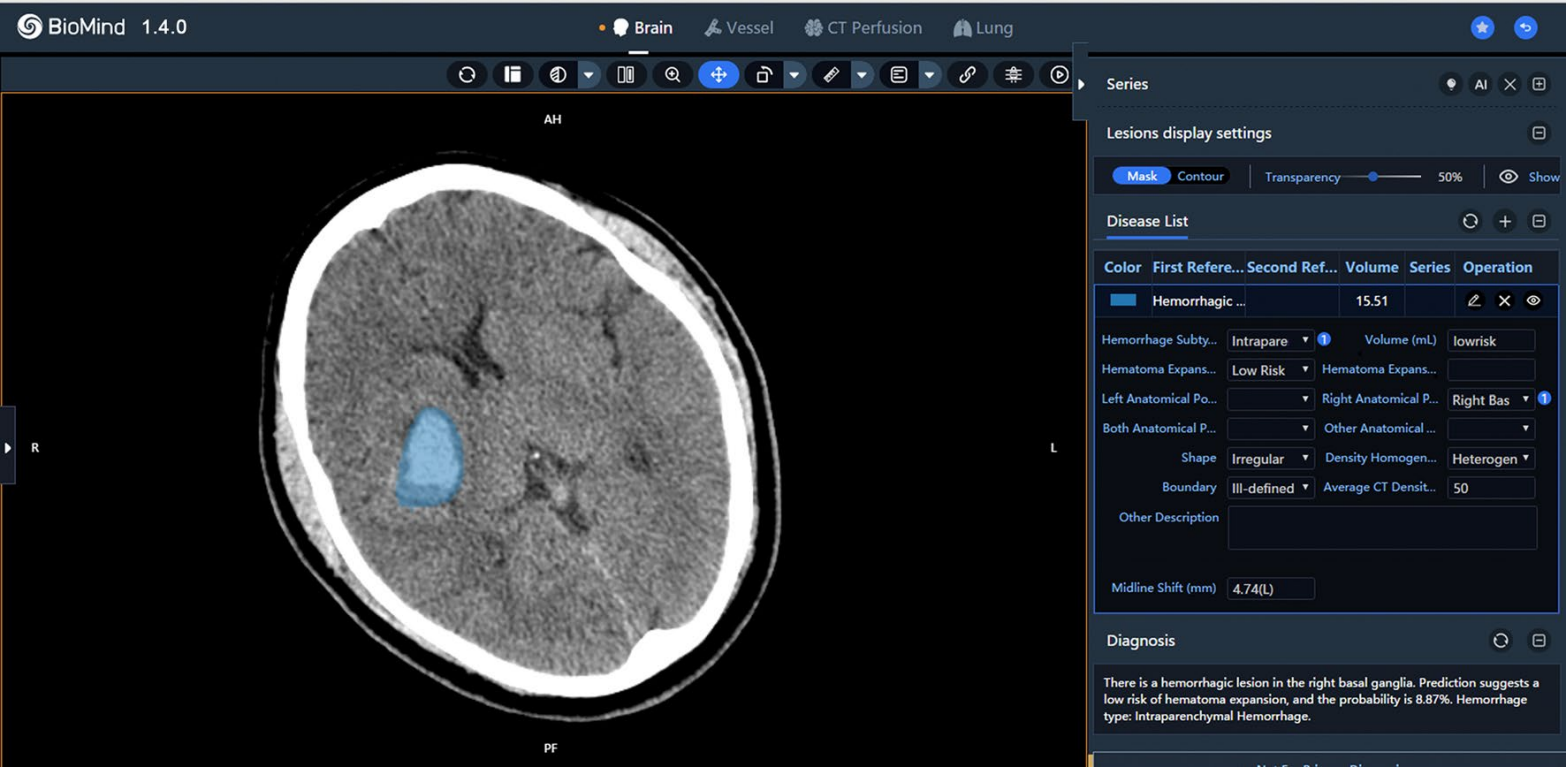
The illustration of AI software user interface using initial CT scan image in predicting hematoma expansion of a 38-year-old patient. (Left) AI software automatically outlined the contour of the hematoma in blue. (Right) The interface of the software output results

Initial CT scan images of the brain were imported to the AI aided diagnostic system (BioMind 1.4.0, Hanalytics, Beijing, China) for automatic hematoma expansion prediction (Fig. 1). The deep learning system was developed by involving 1771 hypertensive intracerebral hemorrhage patients from 84 hospitals spread among 9 provinces and 25 cities, all of which were members of the Chinese Stroke Center Alliance. The proposed model achieved a sensitivity and specificity of 89.3% and 81.1% for hematoma expansion [26].

### Doctor's diagnosis

In our hospital’s practice, an imaging report in the radiology department is generally completed by two radiologists: one radiologist initially writes a report and then the report is reviewed by another radiologist. In order to reduce interpretation variability by two radiologists for predicting hematoma expansion, image interpretation criteria were applied to train the two radiologists with more than 5 years of experience in diagnosing hypertensive intracerebral hemorrhage. Imaging signs for hematoma expansion diagnosis were: (1) Black hole sign: hypoattenuating area encapsulated within the hyperattenuating hematoma with a clearly defined border and the hematoma should have at least 28 Hounsfield unit (HU) difference between the 2 density regions [17]. (2) Blend Sign: blending of the hypoattenuating area and hyperattenuating region with a well-defined margin, the hematoma should have at least 18 HU difference between the 2 density regions [20]. (3) Island Sign: ≥ 3 scattered small hematomas all separate from the main hematoma [5]. Each of the three signs identified in patient CT images confirms hematoma expansion. Initial CT scan images were independently read by two radiologists and they were not aware of the clinical manifestations of the patients and the review results of the CT follow-up scan within 24 h. The hematoma expansion was predicted by analyzing the imaging signs of the initial CT scan. When opinions are inconsistent, the consensus is reached through discussion.

### Statistical analysis

SPSS 27.0 (IBM Armonk, NY, USA) software was used for statistical analysis with *p* < 0.05 as statistical significance. The final diagnosis of the neuroradiologist was used as the gold standard. The sensitivity, specificity and accuracy of the AI group and doctor group in predicting hematoma expansion were compared. Categorical variables between the two groups were compared using the McNemar’s test presented in *p* values. Inter-reader agreement test between the two radiologists were performed, kappa test was applied on these categorical variables. Additionally, AI diagnostic time, doctor’s diagnostic time and gold standard diagnosis time of hematoma expansion was compared. Continuous variables were compared between groups with Student’s *t*-test expressed as the mean ± standard deviation.

## Results

### Patient information

**Fig. 2:**
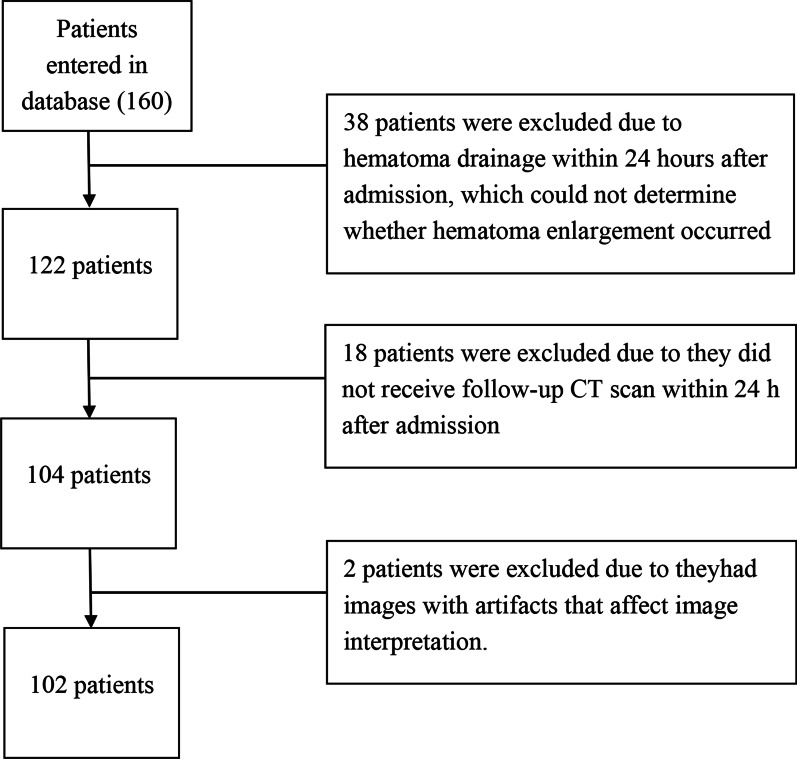
Collection of the retrospective dataset study flow

Inclusion and exclusion criteria for patient recruitment in this retrospective study were summarized in Fig. 2 and a total of 160 patients with HICH were initially collected. 38 patients underwent hematoma drainage within 24 h after admission were excluded since it was impossible to determine whether hematoma expansion occurred during the interval. 18 patients who died after admission or transferred from the hospital did not undergo follow-up CT scan within 24 h and 2 patients had images with artifacts that affect image interpretation were also excluded. A total of 102 cases were finally enrolled including 71 males and 31 females, with an average age of 64.5 ± 13.2 years (age range 32–89 years).

### The predictive result of AI group and doctor group for hematoma expansion in HICH patients

**Fig. 3:**
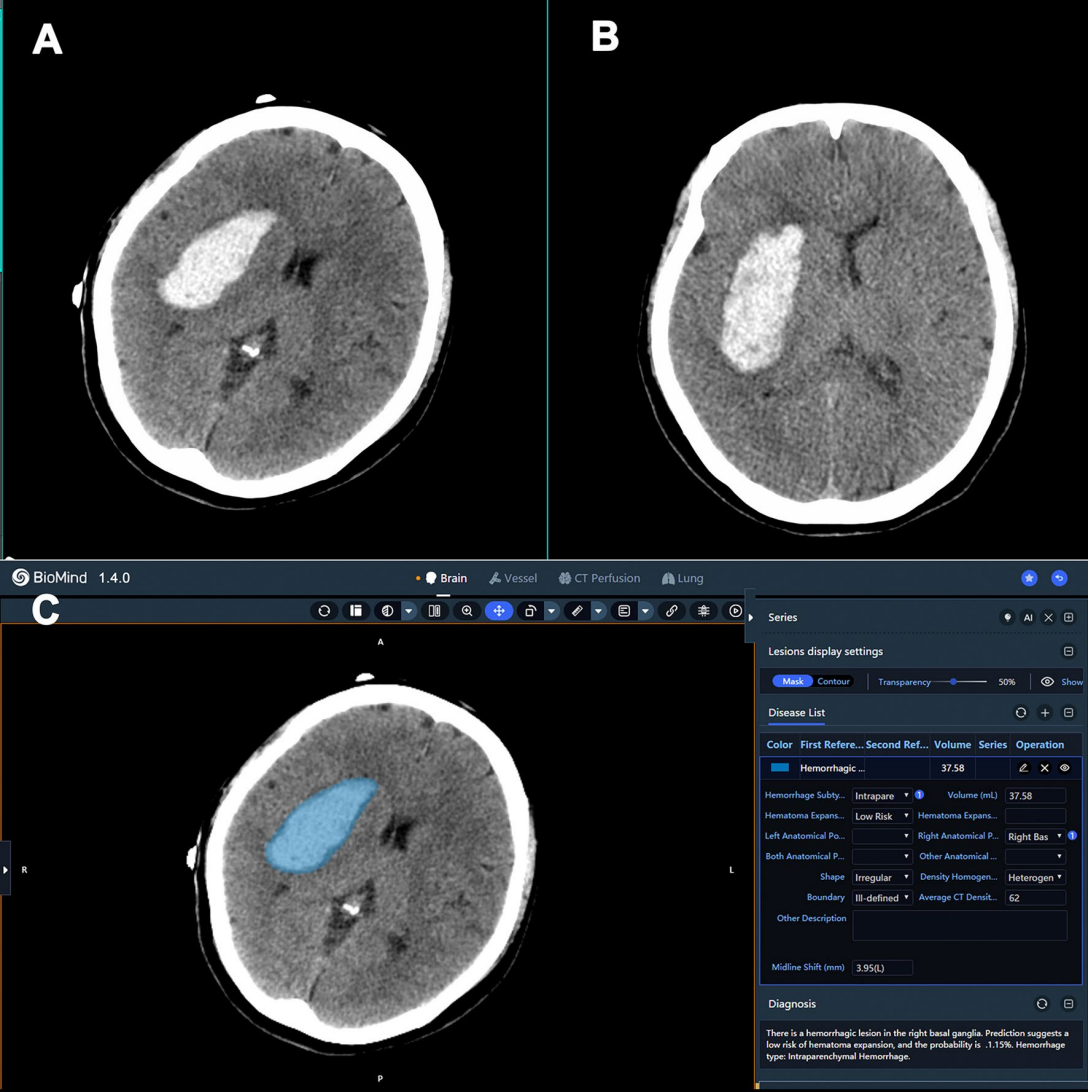
**A** Initial CT scan image of a 51-year-old female HICH patient. The patient was diagnosed with hematoma expansion by the doctor according to CT signs. **B** Follow-up CT scan within 6 h after the initial scan, the hematoma volume increased by 12.9 ml, and was confirmed as hematoma expansion. **C** AI software automatically analyzed the initial CT scan image of the patient after onset. The blue colored mask refers to the delineation of hematoma and the prediction of hematoma expansion by AI software was shown in the right side

**Fig. 4:**
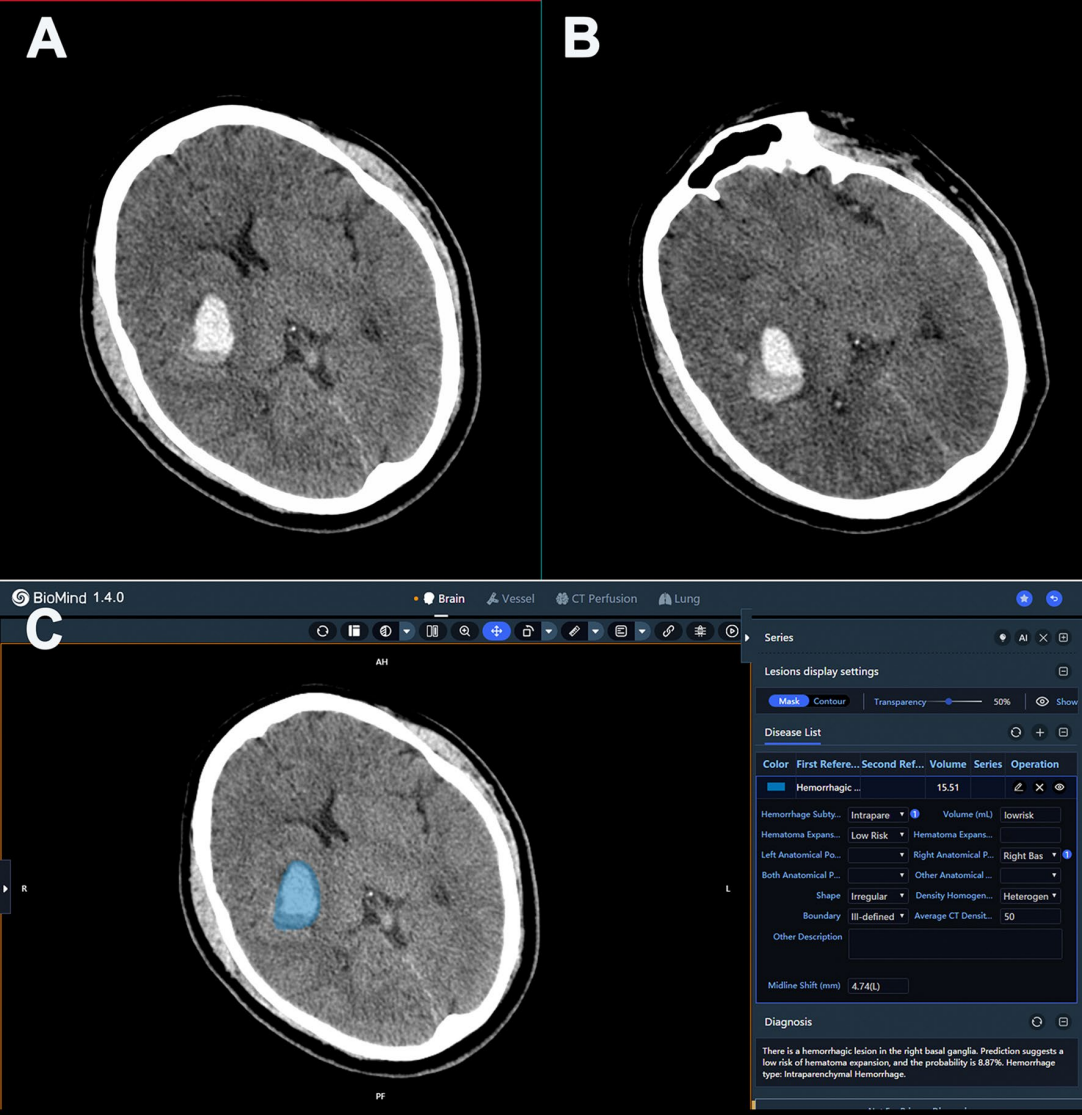
**A** Initial CT scan image of a 38-years-old male HICH patient. The patient was diagnosed with hematoma expansion by doctors based on CT signs. **B** Follow-up CT scan within 12 h after the initial scan. The hematoma volume increased by 2.4 ml and was confirmed by the neuroradiologist as non-hematoma expansion. **C** AI software automatically analyzed the initial CT scan images for hematoma expansion. The patient was diagnosed without hematoma expansion

Among 102 HICH patients, about 29.41% (30/102) patients had hematoma expansion. The prediction sensitivity of the AI group was 80.0% (24/30, 95% CI 0.609–0.916), the specificity was 73.6% (53/72, 95% CI 0.617–0.830) and the accuracy was 75.5% (77/102, 95% CI 0.660–0.835) (Table 1). The prediction sensitivity of the doctor group was 66.7% (20/30, 95% CI 0.471–0.821) the specificity was 58.3% (42/72, 95% CI 0.461–0.696) and the accuracy was 60.8% (62/102, 95% CI 0.506–0.703) (Table 2). The sensitivity (Fig. 3), specificity (Fig. 4) and accuracy of hematoma expansion prediction in the AI group were higher than those in the doctor group (sensitivity: *p* = 0.013; specificity: *p* = 0.043; accuracy: *p* = 0.018). Inter-reader agreement test results between the two radiologists were obtained and the kappa value (κ) was 0.882 which indicated almost perfect agreement between the two radiologists. Kappa values were interpreted as follows: κ $\le \hspace{0.17em}$0 as indicating no agreement and 0.01–0.20 as none to slight, 0.21–0.40 as fair, 0.41–0.60 as moderate, 0.61–0.80 as substantial and 0.81–1.00 as almost perfect agreement.

**Table 1:** Confusion matrix of AI group for hematoma expansion prediction analysis

		Hematoma expansion gold standard		Total
		Positive	Negative	
AI’s diagnosis	Positive	24	19	43
	Negative	6	53	59
Total		30	72	102

**Table 2:** Confusion matrix of doctor group for hematoma expansion prediction analysis

		Hematoma expansion gold standard		Total
		Positive	Negative	
Doctor’s diagnosis	Positive	20	30	50
	Negative	10	42	52
Total		30	72	102

### The diagnosis time of hematoma expansion in AI group and doctor group

The average review time of brain CT images was 14.5 ± 8.8 (hours), while the diagnosis time of AI was 2.8 ± 0.3 s, and the diagnosis time of doctors was 11.7 ± 0.3 s. The diagnosis time of doctors was significantly shorter than that of the gold standard (*p* = 0.001). The diagnosis time of AI was significantly shorter than that of doctors and gold standard (*p* = 0.001). The diagnosis time of AI was more than 99% shortened than that of gold standard, and about 76.3% shortened than that of doctors.

## Discussion

At present, there is no consensus on the definition of hematoma expansion. Some studies defined hematoma expansion as a 33% increase in the relative volume of the hematoma or an absolute volume increase of 6 ml [5, 27]. Some studies defined hematoma expansion as a 33% increase in the relative volume of the hematoma or an absolute volume increase of 12.5 ml [6, 17, 18]. The latter was widely used in clinical settings especially in large-scale clinical trials. In this study, hematoma expansion was defined as the increase of absolute volume > 12.5 ml or relative volume > 33% in two brain CT scan within 24 h after admission.

In 102 HICH patients in this study, about 29.4% (30/102) had hematoma expansion which is consistent with literature reports [19, 28–30] but lower than the reported 35.4% by Chan et al. [6]. The reason might be that 38 HICH patients who conducted hematoma drainage within 24 h due to massive bleeding or broken into the ventricle were excluded which may potentially lowered the incidence rate of hematoma expansion.

At present, several clinical indicators related to hematoma expansion have been identified such as consciousness level, blood pressure and blood glucose [9]. However, the prediction of hematoma expansion by consciousness level is only applicable to patients with mild initial consciousness [9]. Blood pressure and blood glucose are independent predictors of hematoma expansion. Whereas, most patients will undergo antihypertensive and hypoglycemic treatment after admission, the dynamic changes of blood pressure and blood glucose make hematoma expansion prediction impossible.

Many imaging-based methods for deciding hematoma expansion have been proposed and spot sign of CTA has been explored for hematoma expansion prediction [11, 12]. Certain studies have shown that the sensitivity of CTA spot sign to hematoma expansion was about 26.2–73% [10, 13]. In a recent study, the sensitivity of CTA spot sign to predict hematoma expansion was 34.6% [14]. In addition, spot sign often requires patients to undergo brain CTA immediately within the first several hours of onset. It has been shown that the CTA spot sign had the highest sensitivity to predict hematoma expansion if CTA was conducted within 2 h of onset while the sensitivity was only 60.0% [17, 31]. CTA is not the priority for diagnosing HICH because it could be carried out in some areas with insufficient medical resources. Therefore, most HICH patients could not perform CTA within 2 h after onset and CTA scan increased radiation dose and the injection of contrast agent may lead to allergic reactions and renal damage. These reasons limit the widespread applications of CTA [15, 16]. In another study, Fu et al. performed spectral CTA and explored iodine sign to predict hematoma expansion. The sensitivity of iodine sign to predict hematoma expansion was 91.5% which was significantly higher than that of spot sign (61.8%) and iodine sign had a higher accuracy (85.7% vs 75.8%) for hematoma expansion prediction than spot sign [32]. Although the iodine sign had a higher sensitivity and accuracy for hematoma expansion prediction, dual-energy spiral CT is needed and is lacking in areas with insufficient medical resources. Therefore, the use of iodine sign to predict hematoma expansion was not applicable in all medical institutions.

More and more scholars started to predict the expansion of hematoma from the perspectives of the density, shape and the edge of the hematoma on NCCT scan image [5]. Barras et al. [18] studied the brain CT of 90 HICH patients and found that hematoma heterogeneity could be an independent predictor of hematoma expansion. The more heterogeneous the density was, the more likely the hematoma expansion. However, the definition of hematoma heterogeneity was not clear, the judgment of hematoma heterogeneity was subjective and the inter-reader agreement was merely moderate [21]. In several other studies, Li proposed to use "black hole sign", "blend sign", and "island sign" to predict hematoma expansion. The study found that the sensitivity of the above three signs to predict hematoma expansion were 31.9%, 39.3% and 44.7% [5, 17, 20]. In addition, in a study of 200 HICH patients, it was found that the sensitivity of black hole sign to predict hematoma expansion was 33.8% which is lower than that (46.5%) of swirl sign. After multivariate analysis, only the black hole sign was an independent predictor of hematoma expansion [33]. In another study, the sensitivity of the black hole sign and blend sign to predict hematoma expansion were 23.1% and 31.7%, respectively. In a study of 307 HICH patients, Deng [30] found that the island sign had higher specificity than the satellite sign in predicting hematoma expansion (98.1% vs 57.9%) and the satellite sign had a higher sensitivity than the island sign (65.6% vs 45.2%). In general, the sensitivity of NCCT scans alone to predict hematoma expansion is relatively low.

In this study, the sensitivity and specificity of AI software in predicting hematoma expansion were 80.0% and 73.6% respectively, which were significantly higher than the sensitivity (66.7%) and specificity (58.3%) of the doctors using NCCT imaging signs. Prediction accuracy of hematoma expansion is crucial for clinical treatment and in some large randomized controlled trials, the failure to improve patient prognosis by inhibiting hematoma expansion is mainly due to the low accuracy of hematoma expansion prediction [34]. In this study, the accuracy of AI to predict hematoma expansion was 75.5% which was significantly higher than that (60.8%) of the doctors. In addition, the diagnosis time of hematoma expansion is crucial for the treatment [22]. A follow-up brain CT scan is often performed in the clinical workflow within 24 h after admission and hematoma expansion is diagnosed by comparing the volume change of hematoma of two CT scans. The interval time is long, during which the HICH patients may deteriorate rapidly. In this study, the average interval time of brain CT was 14.5 ± 8.8 (hours), the AI diagnosis time was 2.8 ± 0.3 s and the doctor diagnosis time was about 11.7 ± 0.3 s. The AI diagnosis time was significantly shorter than those of doctors and the gold standard diagnosis time (*p* < 0.05), AI diagnosis time was shortened by more than 99% and 76.3% compared with the gold standard time and the doctor diagnosis time, respectively. It could be concluded that AI shortens the diagnosis time of hematoma expansion at an early stage while a high sensitivity specificity and accuracy were maintained. In addition, the AI software based on a deep learning is objective and eliminates inter-reader variability, which is helpful for clinical promotion.

In summary, we have externally validated that AI software based on deep learning algorithm could effectively predict hematoma expansion at an early stage from the initial CT scan images of HICH patients after hemorrhage onset. It has a relatively high sensitivity specificity and accuracy could significantly shorten the determination time of hematoma expansion and helps to guide the selection of clinical treatment options. In addition, this method does not rely on CTA scan and has greater applicability, especially for primary hospitals that lack medical resources and cannot carry out CTA. In addition, this method does not require the injection of contrast agents to avoid adverse reactions caused by contrast agents. The prediction of hematoma expansion by AI software provides a new, accurate, easy to use and fast method for the early prediction of hematoma expansion, which may potentially change the diagnostic process of hematoma expansion.

However, there are several limitations of this study. First, the sample size is relatively small. In the future, the sample size could be expanded, to explore further the prediction efficiency of AI software for hematoma expansion. Secondly, the improvement of AI software for doctors’ diagnostic efficacy could be put into future work.

## Conclusion

Deep learning algorithm could effectively predict hematoma expansion at an early stage from the initial CT scan images of HICH patients after onset with high sensitivity and specificity and significantly shortened diagnosis time, which provides a new, accurate, easy-to-use and fast method for the early prediction of hematoma expansion.

## Data Availability

All data generated or analyzed during this study are included in this published article.

## Abbreviations

NCCT: Noncontrast computed tomography
HICH: Hypertensive intracerebral hemorrhage
CT: Computed tomography
AI: Artificial intelligence
SICH: Spontaneous intracerebral hemorrhage
CTA: Computed Tomography Angiography
